# Socioeconomic Status Predicts Short-Term Emergency Department Utilization Following Supratentorial Meningioma Resection

**DOI:** 10.7759/cureus.24508

**Published:** 2022-04-26

**Authors:** Michael Spadola, Ali S Farooqi, Austin J Borja, Ryan Dimentberg, Rachel Blue, Kaitlyn Shultz, Scott D McClintock, Neil R Malhotra

**Affiliations:** 1 Department of Neurosurgery, University of Pennsylvania Perelman School of Medicine, Philadelphia, USA; 2 Department of Mathematics, West Chester University, West Chester, USA

**Keywords:** social determinants of health, readmissions, outcomes, disparity, brain tumor

## Abstract

Introduction

By identifying drivers of healthcare disparities, providers can better support high-risk patients and develop risk-mitigation strategies. Household income is a social determinant of health known to contribute to healthcare disparities. The present study evaluates the impact of household income on short-term morbidity and mortality following supratentorial meningioma resection.

Methods

A total of 349 consecutive patients undergoing supratentorial meningioma resection over a six-year period (2013-2019) were analyzed retrospectively. Primary outcomes were unplanned hospital readmission, reoperations, emergency department (ED) visits, return to the operating room, and all-cause mortality within 30 days of the index operation. Standardized univariate regression was performed across the entire sample to assess the impact of household income on outcomes. Subsequently, outcomes were compared between the lowest (household income ≤ $51,780) and highest (household income ≥ $87,958) income quartiles. Finally, stepwise regression was executed to identify potential confounding variables.

Results

Across all supratentorial meningioma resection patients, lower household income was correlated with a significantly increased rate of 30-day ED visits (p = 0.002). Comparing the lowest and highest income quartiles, the lowest quartile was similarly observed to have a significantly higher rate of 30-day ED evaluation (p = 0.033). Stepwise regression revealed that the observed association between household income and 30-day ED visits was not affected by confounding variables.

Conclusion

This study suggests that household income plays a role in short-term ED evaluation following supratentorial meningioma resection.

## Introduction

The social determinants of health (SDOH) refer to the environmental, social, economic, and cultural factors, such as gender, race, socioeconomic status (SES), and education level, outside of the immediate medical setting, which impact a patient’s quality of health. The medical community has increasingly focused on the contribution of SDOH to healthcare disparities. Further, policies have been introduced that emphasize value-based care models, incentivizing the elimination of SDOH-related disparities to reduce avoidable costs.

In the surgical setting, SES has been previously demonstrated to predict postoperative outcomes across a wide range of populations. Within neurosurgery, previous studies have shown that low SES puts patients at risk for complications following multiple different procedures, from brain tumor resection to spinal surgery [1-5]. Given its broad impact, it, therefore, remains important to evaluate SES and identify outcome disparities in specific neurosurgical procedural populations.

Here, we evaluate the impact of SES on outcomes following supratentorial meningioma resection. Supratentorial meningiomas account for nearly half of all primary, non-malignant intracranial lesions [6]. Further, in contrast to other cranial tumors, supratentorial meningiomas have favorable histology and significant long-term survival with maximal safe resection [7-8]. Nonetheless, surgical resection bears an appreciable complication profile, including neurological deficits and seizures [9-10]. As such, the identification of patient characteristics that drive outcome disparities in this population is essential for developing risk-mitigation strategies.

Previous studies in meningioma patients have shown that lower SES is correlated with a decreased likelihood of resection and worse overall survival [11-12]. Further, the present authors observed that meningioma patients with lower household income experienced higher rates of Emergency Department evaluation within 90 days of resection [13]. However, few studies have evaluated morbidity and mortality specifically within the 30-day postoperative window. This time frame is meaningful to consider, as 30-day surgical outcomes are incorporated into multiple hospital grading scales and reimbursement models. The objective of the present study is to assess the effect of SES, specifically household income, on 30-day supratentorial meningioma resection outcomes.

## Materials and methods

Sample selection

**Figure 1:**
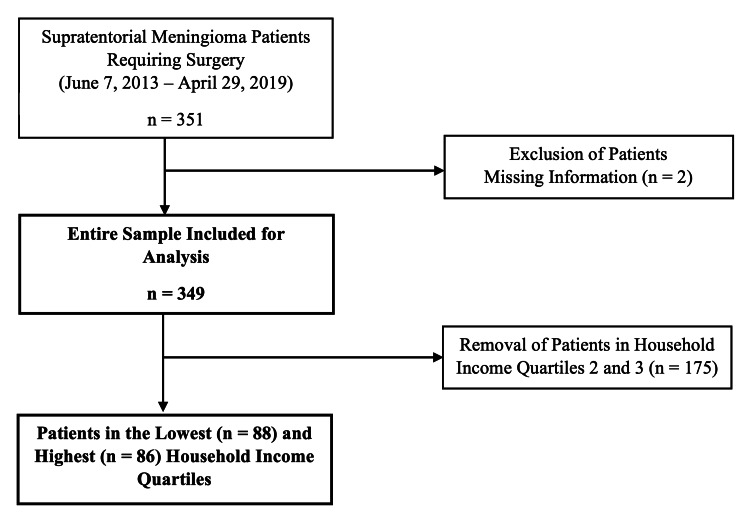
Patient Selection Flowchart describing the selection of supratentorial meningioma cases across a six-year period

This study retrospectively enrolled 349 consecutive patients with complete health information who underwent supratentorial meningioma resection at a multi-hospital, 1659-bed university health system in Philadelphia, PA, USA, over a six-year period (June 7, 2013 - April 29, 2019) (Figure 1), as previously described by the present authors [13]. Data were acquired using the EpiLog tool - a non-proprietary data acquisition system layered on top of the electronic health record at the present institution to facilitate charting, workflow, quality improvement, and cost reduction initiatives [14].

Data collection and statistical analysis

Patient characteristics and outcome data were extracted via EpiLog and pushed into defined spreadsheets. Recorded patient characteristics included age, race, gender, body mass index (BMI), zip code, American Society of Anesthesiologists (ASA) score, tobacco use within 12 months prior to surgery, Charlson Comorbidity Index (CCI) score, prior surgical history, total duration of surgery, total cost, and level of education. Household income was determined by cross-referencing patient zip codes with demographic data from the 2012-2016 U.S. Census Bureau 5-Year American Community report [15].

Outcomes included unplanned hospital readmission, reoperations, ED visits, return to the operating room, and all-cause mortality within 30 days of the index operation. Standardized univariate logistic regression was carried out across the entire sample to assess the impact of increasing household income on outcomes. Odds ratio (OR) < 1 indicates that the outcome was more likely with lower household income. Subsequently, patients were separated into quartiles based on household income, and univariate regression was repeated to compare outcomes between the lowest and highest income quartiles. OR < 1 indicates that the outcome was more likely in the lowest income quartile. Finally, a stepwise regression model, incorporating other recorded patient demographic variables, was used to identify potential confounders. For outcomes that had less than 5% of events occur (30-day reoperation and mortality), a Firth correction was applied to eliminate the small sample size bias This statistical analysis has previously been described by the present authors [1,5,13]. Significance for all analyses was set at a p-value < 0.05.

## Results

Patient characteristics

Across all patients (n = 349), the mean age was 58.9 ± 14.2 years, mean BMI was 28.9 ± 6.3, and mean CCI score was 2.78 ± 2.33 (Table 1). Further, 63.0% were female and 12.6% reported prior tobacco use. The average household income was $70,608, ranging from $18,119 to $191,354.

**Table 1:** Patient Characteristics Patient demographics and baseline characteristics across the entire sample (n = 349), as well as between the lowest (Q1) and highest (Q4) household income quartiles sd=standard deviation

Variable	Entire Sample (n = 349)	Q1 (n = 88)	Q4 (n = 86)	Standardized Difference
Age, years, mean (sd)	58.9 (14.2)	57.0 (15.4)	60.9 (13.5)	0.27
Gender, n (%)				0.11
Male	129 (36.96)	32 (36.36)	36 (41.86)	
Female	220 (63.04)	56 (63.64)	50 (58.14)	
Race, n (%)				1.26
Asian	8 (2.29)	2 (2.27)	2 (2.33)	
Black/African American	63 (18.05)	38 (43.18)	3 (3.49)	
White	250 (71.63)	38 (43.18)	77 (89.53)	
Hispanic/Latino	10 (2.87)	5 (5.68)	2 (2.33)	
Other	18 (5.16)	5 (5.68)	2 (2.33)	
Tobacco Use within Past 12 Months, n (%)				0.29
Yes	44 (12.61)	14 (15.91)	7 (8.14)	
No	292 (83.67)	71 (80.68)	78 (90.70)	
Unknown	13 (3.72)	3 (3.41)	1 (1.16)	
Body Mass Index, kg/m^2^, mean (sd)	28.9 (6.3)	30.0 (6.8)	27.5 (5.5)	-0.40
American Society of Anesthesiologists Grade, n (%)				0.60
1	0 (0)	0 (0)	0 (0)	
2	131 (37.54)	19 (21.59)	42 (48.84)	
3	209 (59.89)	66 (75.00)	42 (48.84)	
4	7 (2.01)	3 (3.41)	2 (2.33)	
Charlson Comorbidity Index Score, mean (sd)	2.78 (2.33)	2.88 (2.52)	2.74 (2.08)	-0.06
Surgeries within 90 Days Prior to Index Operation, n (%)				-0.07
0	331 (94.84)	85 (96.59)	84 (97.67)	
1	17 (4.87)	3 (3.41)	2 (2.33)	
2+	1 (0.29)	0 (0)	0 (0)	
Lifetimes Surgeries Prior to Index Operation, n (%)				0.29
0	326 (93.41)	79 (89.77)	82 (95.35)	
1	13 (3.71)	5 (5.68)	3 (3.49)	
2+	8 (2.29)	4 (4.56)	1 (1.16)	
Length of Stay, hours, mean (sd)	125.1 (133.8)	150.2 (158.4)	126.9 (149.1)	-0.15
Total Cost, $, mean (sd)	3054.37 (1528.88)	3272.76 (1609.66)	2940.17 (1373.36)	-0.22
Duration of Surgery, minutes, mean (sd)	217.1 (117.5)	241.1 (131.1)	214.8 (124.3)	-0.21

Patients in the lowest income quartile (Q1) had an income range from $18,119 - $51,780, while patients in the highest quartile (Q4) had an income range from $87,958 - $191,354. Additionally, there were more black/African American patients in Q1 (43%) than Q4 (3%) (Table 1).

Patient outcomes

**Figure 2:**
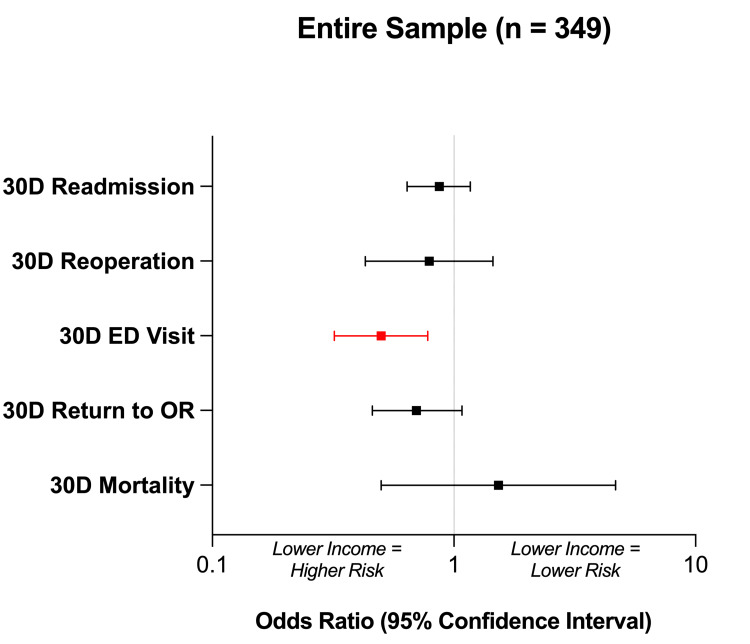
Entire Sample Outcomes Forest plot demonstrating standardized univariate logistic regression across the entire sample (n = 349) to assess the impact of increasing household income on outcomes. An odds ratio of < 1 indicates that the outcome was more likely with a lower household income. Red values denote statistical significance at p < 0.05.

Across all patients (n = 349), univariate analysis revealed a significant, negative correlation between household income and 30-day rate of ED evaluation (p = 0.002, OR = 0.50) (Table 2, Figure 2). Further, a trend was observed between lower household income and increased 30-day return to the operating room; however, this trend was not statistically significant (p = 0.10, OR = 0.70). No associations were seen between household income and 30-day readmission (p = 0.36) or reoperation (p = 0.44). Across all patients, there was only a single recorded mortality event within 30 days (overall rate 0.29%). No correlation was demonstrated between household income and 30-day mortality (p = 0.46).

**Table 2:** Patient Outcomes Standardized univariate logistic regression was carried out on the entire sample (n = 349) to assess the impact of increasing household income on outcomes (left columns). Subsequently, univariate logistic regression was executed to compare outcomes between the lowest (Q1; n = 88) and highest (Q4; n = 86) household income quartiles (right columns). An odds ratio of < 1 indicates that the outcome was more likely with lower household income. Bolded values denote statistical significance at p < 0.05. CI=confidence interval, ED=emergency department, OR=odds ratio

Outcome	Entire Sample			Q1 vs Q4			
	n (%)	OR (95% CI)	P-value	Q1, n (%)	Q4, n (%)	OR (95% CI)	P-value
30-Day Readmission	56 (16.05)	0.87 (0.64-1.17)	0.36	19 (21.59)	14 (16.28)	0.71 (0.33-1.52)	0.37
30-Day Reoperation	12 (3.44)	0.79 (0.43-1.45)	0.44	4 (4.55)	2 (2.33)	0.56 (0.20-1.69)	0.47
30-Day ED Visit	33 (9.46)	0.50 (0.32-0.78)	0.002	13 (14.77)	4 (4.65)	0.28 (0.09-0.90)	0.033
30-Day Return to the Operating Room	29 (8.31)	0.70 (0.46-1.08)	0.10	10 (11.36)	6 (6.98)	0.59 (0.20-1.69)	0.32
30-Day Mortality	1 (0.29)	1.53 (0.50-4.67)	0.46	0 (0)	0 (0)	N/A	N/A

**Figure 3:**
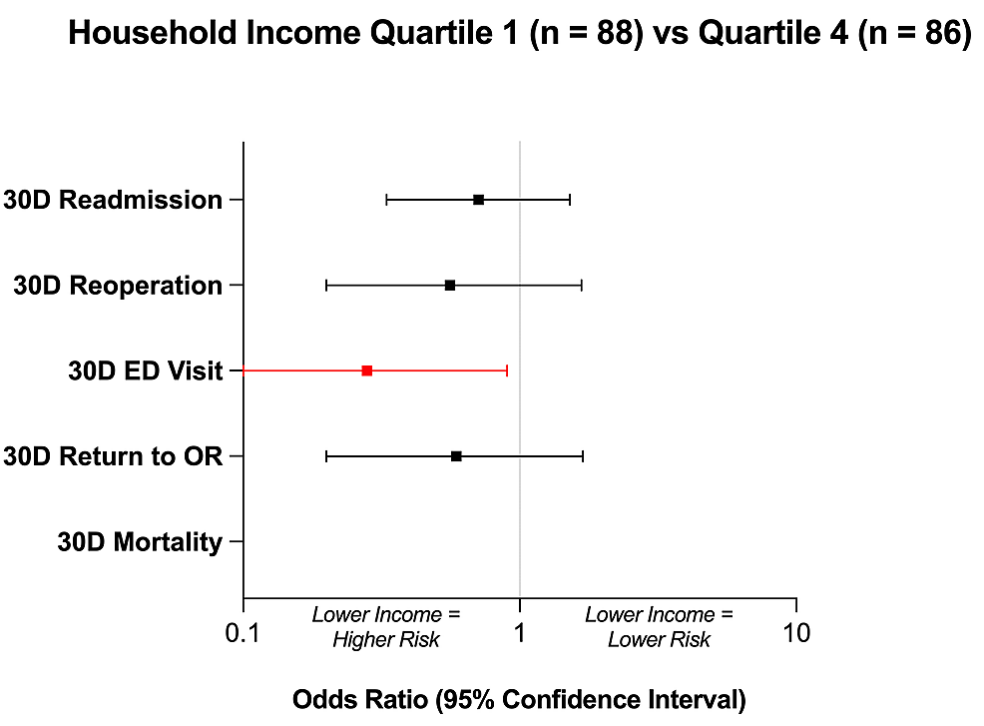
Household Income Quartile Outcomes Forest plot comparing outcomes between the lowest (Quartile 1; n = 88) and highest (Quartile 4; n = 86) household income quartiles. An odds ratio of < 1 indicates that the outcome was more likely in the lowest household income quartile. Red values denote statistical significance at p < 0.05.

Comparing Q1 and Q4, the lowest income quartile was revealed to have a significantly increased 30-day rate of ED evaluation (p = 0.033, OR = 0.28) (Table 2, Figure 3). However, no differences in 30-day readmission (p = 0.37), reoperation (p = 0.47), or return to the operating room (p = 0.32) rates were observed between Q1 and Q4. No mortality events occurred in Q1 or Q4, so an inter-quartile comparison of 30-day mortality was not conducted.

Stepwise regression

The observed association between household income and 30-day ED evaluation was not affected by any confounding variables. Conversely, the 30-day readmission rate was confounded by education level and CCI score. Additionally, the 30-day reoperation rate was confounded by CCI score, and the 30-day rate of return to the operating room was confounded by race, CCI score, and ASA grade. Finally, the 30-day mortality rate was affected by a history of prior surgery within 90 days.

## Discussion

In this study, we assessed the impact of household income on short-term morbidity/mortality measures across 349 consecutive supratentorial meningioma resection patients. In both the entire sample and inter-quartile analyses, lower income was significantly correlated with increased ED visits within 30 days of the index operation. No additional associations were observed between household income and our other primary outcomes. Finally, only one mortality event was recorded within the 30-day postoperative window, with no mortalities in either the Q1 or Q4 subgroup, consistent with previously published meningioma outcome data indicating rates of 30-day mortality less than 10% [16-17].

The present authors previously observed that lower household income predicted increased 90-day ED evaluation following supratentorial meningioma resection [13]. In contrast to the previous study, here we focused our analysis on the 30-day window following the index operation. This time frame was an intentional feature of our study design, as outcomes within 30 days of surgery may be factored into surgical reimbursement and hospital ratings. Our study demonstrates that household income independently predicts short-term ED evaluation following meningioma resection. This finding may be explained by several factors, including differences in insurance status, geographic barriers to outpatient care facilities, and social factors (including diet or substance use/abuse) [18-19]. For instance, low-income and uninsured patients often rely on emergency facilities for primary evaluation, which may be reflected in our results [20]. In contrast, household income was not correlated to other adverse events following supratentorial meningioma resection. These negative findings may reflect the comparably low complication rate and favorable long-term prognosis in this population [21-22]. Meningiomas are characterized by their benign pathology and slow progression, which lead to favorable morbidity and mortality postoperatively [23-24].

The results of the present study are immediately applicable to patients, providers, and healthcare systems. Moving forward, strategies are needed to identify and support high-risk meningioma patients. Household income, and other important patient characteristics, may be incorporated into models to predict adverse outcomes [25]. Further, tailored preoperative education, regularly scheduled outpatient visits, and immersive social work involvement may help reduce ED utilization, as well as curb healthcare costs [26-28]. Future, prospective work ought to leverage our findings to mitigate income-related healthcare disparities.

Limitations

One limitation is that this study is retrospective, opening the possibility of sampling bias and data inaccuracies. Further, outcome data were collected via the electronic medical record, potentially underreporting adverse events that occurred outside the primary health system. However, all patients received follow-up beyond the 30-day postoperative window (median follow-up of 700 days), and during each outpatient visit, patients were asked about encounters at other health systems. Also, any discrepancies would be expected to be consistent across all subjects, maintaining the internal validity of our study.

Another limitation is that this study indirectly extracted household income from patient zip code instead of directly recording patient income status. Nonetheless, previous studies have demonstrated that zip-level median household income is effective for detecting health outcome differences [29].

Finally, other important patient characteristics may confound the present results. Here, we utilized stepwise analysis to identify such confounders among multiple recorded patient characteristics known to impact surgical outcomes. We observed that the 30-day rate of ED evaluation was not confounded by any other variables, indicating a robust and independent correlation. Nonetheless, further studies using matched cohorts may better isolate the relationship between income and outcomes to corroborate the present findings.

## Conclusions

By identifying patient characteristics that underlie outcome disparities, providers can better support the highest-risk patients. Our results suggest that household income can predict ED utilization within the short-term postoperative window following supratentorial meningioma resection. Future, dedicated studies are needed to examine the impact of income on other neurosurgical populations, as well as to develop interventions that improve patient outcomes and eliminate income-related healthcare disparities.
